# Impact of the 2010 Deepwater Horizon oil spill on population size and genetic structure of horse flies in Louisiana marshes

**DOI:** 10.1038/srep18968

**Published:** 2016-01-12

**Authors:** Claudia Husseneder, Jennifer R. Donaldson, Lane D. Foil

**Affiliations:** 1 Department of Entomology, Louisiana State University Agricultural Center, Baton Rouge, LA 70803

## Abstract

The greenhead horse fly, *Tabanus nigrovittatus* Macquart, is frequently found in coastal marshes of the Eastern United States. The greenhead horse fly larvae are top predators in the marsh and thus vulnerable to changes in the environment, and the adults potentially are attracted to polarized surfaces like oil. Therefore, horse fly populations could serve as bioindicators of marsh health and toxic effects of oil intrusion. In this study, we describe the impact of the April 2010 Deep Water Horizon oil spill in the Gulf of Mexico on tabanid population abundance and genetics as well as mating structure. Horse fly populations were sampled biweekly from oiled and unaffected locations immediately after the oil spill in June 2010 until October 2011. Horse fly abundance estimates showed severe crashes of tabanid populations in oiled areas. Microsatellite genotyping of six pristine and seven oiled populations at ten polymorphic loci detected genetic bottlenecks in six of the oiled populations in association with fewer breeding parents, reduced effective population size, lower number of family clusters and fewer migrants among populations. This is the first study assessing the impact of oil contamination at the level of a top arthropod predator of the invertebrate community in salt marshes.

Coastal salt marshes are among the most valuable, productive and diverse ecosystems on earth^1^. Critical ecosystem services provided by salt marshes include coastal protection, nutrient production and cycling, support and shelter for an extraordinary food web and biodiversity, sustaining the harvest of seafood and recreational activities. Coastal marshes are also among the most vulnerable ecosystems threatened by natural as well as man-made disasters. Oil spills in particular have added to the degradation of marshes in the recent past, causing immediate death of plants and animals as well as long-term effects, such as increased erosion, persistence of oil-derived components in the food web and overall decrease in ecosystem services [2 and references therein].

To date, the largest accidental man-made marine oil spill was the catastrophic explosion of the Deep Water Horizon drilling platform connected to the blowout of British Petroleum’s Macondo well, which occurred on April 20, 2010 and resulted in 4.9 million barrels of oil discharged into the Gulf of Mexico over a period of almost four months^3^,^4^. The salt marshes of East Louisiana took the brunt of the oil spill with an estimated 75 linear km with reported moderate to heavy oiling [2 and references therein].

Acute oiling in the Gulf of Mexico and the coastal habitats subsequent to the Deepwater Horizon platform collapse created visible impact on highly prominent vertebrates, particularly birds, marine mammals and turtles along the coast^5^. Subsequent to the spill, subsurface water and sediments in Louisiana marshes were shown to have significant levels of oil residues and the effects on fish ranged from acute toxicity to genomic and physiological changes that were detectable for months after the oil spill^6^,^7^.

Contamination is known to alter soil properties and impact the food web from microbes to plants and invertebrate populations and vertebrates that use the marsh for feeding and nursery areas^8^. These observations underscore the importance for monitoring the impact of oil spills on the health of salt marsh ecosystems in a cost-efficient way with minimum impact on the ecosystem.

The salt marsh insect community is the trophic support for both terrestrial and marine vertebrates^9^ and herbivourous salt marsh artropod communities were shown to be vulnerable to oil exposure^10^. Aquatic insects have been frequently used as biological barometers of status of freshwater ecosystems biodiversity and also as bioaccumulators of metals and other contaminants^11^,^12^. However, studies on insect biology in brackish and saline ecosystems are relatively rare because there are few insect species that are osmotolerant, and the literature of use of insects in biomonitoring programs for tidal marsh integrity, ecotoxicological studies, or bioaccumulation of pollutants is scant^13^.

We evaluated populations of the greenhead horse fly, *Tabanus nigrovittatus* Macquart (Diptera: Tabanidae) as a bioindicator of the impact of the intrusion of oil into coastal marshes because this horse fly is one of very few insects that are native to and tightly bound to specific coastal marsh habitats that range from the Texas coast to Nova Scotia^14^. The greenhead horse fly larvae develop for 3–9 months and are top predators of invertebrates in the marsh soil^15^. Their abundance is thus reflective of the health of the invertebrate food web around them in the mud of the tidal marshes.

Our overall hypothesis was that the oil residue impacts the invertebrate ecosystems in tidal marshes, and that tabanid residents of the marsh community would serve as bioindicators for wetland health and the oil intrusion in particular, as they require both aquatic and terrestrial habitat for survival. To test this hypothesis, we sampled adult horse fly populations biweekly immediately after the oil spill (June 2010-October 2011) via canopy traps from oiled areas and those unaffected by oil (Fig. 1). We also collected larvae from the marsh soil. First, we compared population abundance based on a census of horse flies between unaffected areas of the marsh and those that have been affected by the oil spill. We also used previously developed microsatellite loci of *T. nigrovittatus*^16^ to compare population genetic parameters (e.g., population structure, recent bottlenecks and migration) and mating structure between populations from unaffected and contaminated areas. By combining population census data and population genetic analysis this study sheds light on the fate of populations of this top predator in Louisiana marshes after the oil spill.

## Results

### Lower abundance of horse fly adults and larvae in oiled areas

**Figure 1:**
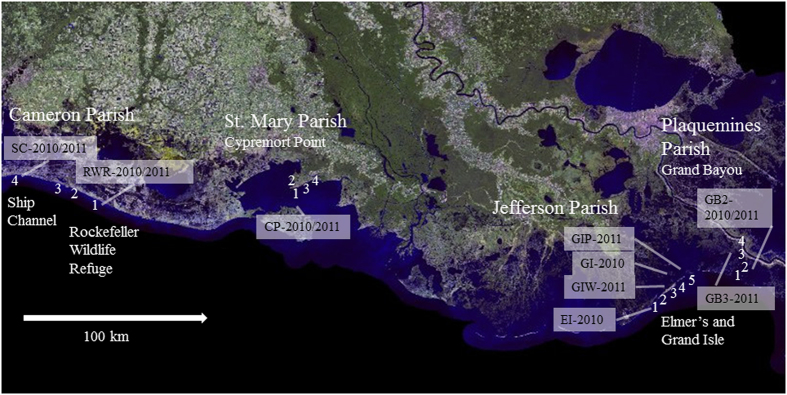
Map of the sampling locations of unaffected (Cameron and St. Mary Parish in West Louisiana) and oiled (Jefferson and Plaquemines Parish in East Louisiana) populations of tabanids. Collections from four locations with 4–5 adult collection sites (traps) were used for population abundance studies. Samples also used for population genetic analyses are marked by text boxes (SC = Ship Channel, RWR = Rockefeller Wildlife Refuge, CP = Cypremort Point, EI = Elmer’s Isle, GI = Grand Isle, GIW = Grand Isle West, GIP = Grand Isle Park, GB = Grand Bayou). Map based on Landsat 2005 imagery available from Louisiana Department of Natural Resources Strategic Online Natural Resources Information System (SONRIS) [ http://sonris-www.dnr.state.la.us/gis/agsweb/IE/JSViewer/index.html?TemplateID=181, date of access: 11/05/2015]. Sample sites were added using PowerPoint 2010.

Our trap data with only a range of 1.3–4.8 flies captured per hour in 41 trap days indicated that the adult tabanids had been affected immediately after the oil reached Elmer’s Isle, Grand Isle (Jefferson Parrish) and Grand Bayou (Plaquemines Parish, Fig. 1); both of these areas were notorious for greenhead attack each summer. In comparison, fly activity remained high at the unaffected locations at Cypremort Point (St. Mary Parish) and Cameron Parish (a range of 36.6 – 92.2 flies per hour in 60 trap days).

**Figure 2:**
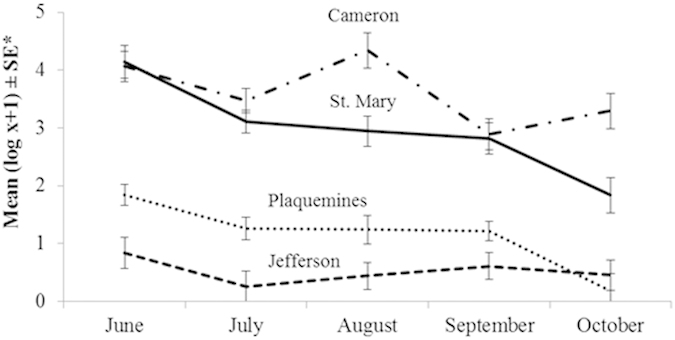
Seasonal fluctuation of adult *T. nigrovittatus* caught in unaffected (Cameron, St. Mary) and oiled regions (Plaquemines, Jefferson) averaged across years.

At each of the four regions the mean number of flies per hour trapped was not significantly different between the years 2010 and 2011 (Table 1). In 2010, horse fly numbers differed significantly (P = 0.0042) between all four regions, while in 2011 catches at the two unaffected locations (Cameron, St. Mary) were equally high and catches at oiled locations (Jefferson, Plaquemines) were equally low. Overall, in both years, horse fly numbers caught at unaffected locations were significantly higher (P < 0.0001) than at oiled locations (Table 1). As expected, the number of trapped flies fluctuated seasonally at each location (Fig. 2), but counts at oiled locations were in most cases by magnitudes lower than those at unaffected locations (Table S1, in Supporting Information).

In support of the observed impact of oil intrusion on adult horse flies, we recorded lower incidence rates of larva recovery from oiled areas. At Grand Isle, no larvae were collected from any of the six collection sites and there was a distinct black residue in most of the sediment samples. At Grand Bayou, 1 tabanid larva was isolated from only 1 of 8 sediment samples. In addition, only four other immature insects that were not tabanids were isolated from 4 of the 8 sediment samples.

In contrast to oiled areas, there was a high probability of collecting tabanid larvae in those spartina marshes in Louisiana that were not affected by the oil spill. Tabanid larvae were isolated from 4 of the 5 samples obtained in Cameron; the maximum number of larvae was 10 with an average of 3 per sample. From Cypremort Point, larvae were isolated from 5 of the 8 samples; the greatest number of larvae was 2 with an average of 1. We also found 11 other immature insects (not tabanids) in 3 of the 13 sediment samples from the areas unaffected by oil.

### Population differentiation, genetic diversity and isolation by distance

All samples across two years from the trap sites selected for genotyping were significantly differentiated based on their allele frequencies at the 1/1000 level (78,000 permutations, FSTAT). Thus, the 13 samples were treated as genetically separated populations. Summary statistics for each locus and population (allele numbers, inbreeding coefficients and heterozygosity) are listed in Table S2. Hierarchical *F*-statistics confirmed population genetic differentiation among samples (*F*_ST_ = 0.32, *SE* = 0.042) and revealed a considerable amount of inbreeding in the total population (*F*_IT_ = 0.52, *SE* = 0.08), and within populations (*F*_IS_ = 0.29, *SE* = 0.04). The local inbreeding coefficient *F*_IS_ was positive in all 13 populations, with values significantly or marginally greater than zero in 7 populations (Table S2), but not significantly different between oiled and non-oiled populations (Mann-Whitney U-test, P > 0.20).

**Figure 3:**
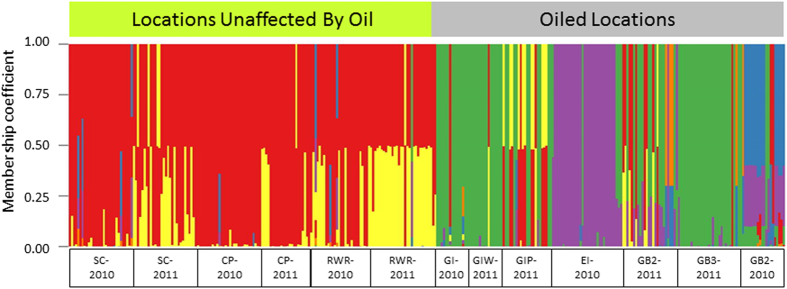
Assignment of adult tabanids individuals from 13 populations (x-axis) collected in 2010 and 2011 from unaffected and oiled locations to six major genetic clusters. The height of colored bars in each column represents the membership coefficient, i.e. the likelihood with which an individual is assigned to each genetic cluster.

TESS analysis assigned the individuals from the 13 sample populations to six major genetic clusters (Figure S1 in Supporting Information). The plot of the membership coefficients that assign each individual to its predominant genetic cluster(s) visualizes a higher degree of admixture within and a higher gene flow among the unaffected populations compared to the majority of the oiled populations. Interestingly, two of the oiled populations in 2011 (GIP-2011, GB2-2011) showed an influx of individuals belonging to genetic clusters (“red” and “yellow” in Fig. 3) that were not predominant in the Eastern populations in 2010, but were characteristic for all Western populations of 2010 and 2011.

Condition (i.e., whether a population originated from unaffected or oiled areas) had marginally significant effects on genetic distance (*F*_ST_) among populations (GLM, *F* = 4.06, *df* = 1, *P* = 0.069, Table S3 in Supporting Information). Year of collection (2010 vs. 2011) had no significant effect, however, there was significant interaction between condition and year (*F* = 9.844, *df* = 1, *P* = 0.009). Since variances were unequal (Levene’s, *F* = 6.39, *df = *3,11, *P* = 0.009), additional *t*-test were performed not assuming equal variances. *T*-tests confirmed marginally lower genetic distance between populations not reached by oil (*mean F*_ST_ = 0.17, *SD* = 0.10) compared to oiled populations (*mean F*_ST_ = 0.23, *SD* = 0.06) and no difference between collection years.

Bayesian analyses showed that recent (over the last few generations) migration rates among populations ranged from 0.1 to 28% with proportions of 68–99% being derived from the source populations each generation (Table S4 in Supporting Information). The emigration rate among unaffected populations (0.014, *SD* = 0.014) was marginally higher than that of oiled populations (0.009, *SD* = 0.004, *P* = 0.07, Mann-Whitney-U), while the import rate was not different (*P* > 0.20). The largest donor among unaffected populations was CP-2010 with gene flow into SC-2010 (0.28) and RWR-2010 (0.11). The largest donor among oiled populations was GIW-2011 with export to GIP-2011 (0.11) and GB3-2011 CP (0.26). Migration rates from unaffected to oiled areas and *vice versa* were overall low and no directional differences were found (unaffected to oiled: 0.005 *SD* = 0.011; oiled to unaffected 0.009, *SD* = 0.042). The low migration rate was supported by the fact that genetic distances between unaffected and oiled populations (*F*_ST_* = *0.39, *SD* = 0.10) were significantly larger than among either unaffected (*F*_ST_* = *0.17, *SD* = 0.10) or oiled (*F*_ST_* = *0.23, *SD* = 0.06) populations likely due to the geographic separation (*P* < 0.001). The only migration between unaffected and oiled areas with a rate exceeding 5% was recorded from SC-2011 to GB2-2011 (0.07, Table S4).

Weak, but significant isolation by distance (i.e., positive correlation between geographical and genetic distance) was found only over a large scale up to 360 km between the Western unaffected populations and the Eastern oiled populations (Mantel test, *Z* = 47.02, *R*^*2*^ = 0.13, *P* = 0.001). No isolation by distance was detected among populations within 150 km (non-oiled: *R*^*2*^ = 0.06, *P* > 0.20, oiled: *R*^*2*^ = 0.01, *P* > 0.20, Figure S2 in Supporting Information).

### Genetic bottlenecks detected in oiled populations

We did not detect recent genetic bottlenecks in any of the six populations collected in 2010 and 2011 that were not impacted by the oil spill under any of the three mutation models (IAM, TPM, SMM). Instead of detecting heterozygote excess as a signature of a recent bottleneck, there was significant heterozygote deficiency detected by at least one mutation model in all but one (RWR-2010) of the unaffected populations, which might indicate a recent population expansion in unaffected areas.

In contrast, we detected significant recent genetic bottlenecks in 2010 and/or 2011 in five out of seven populations from areas that were hit by the oil spill (GI-2010, EI-2010, GB2- and GB3-2011, and GB2-2010, Table 2) and an additional population showed a marginal bottleneck effect (GIP-2011). All populations with signs of bottlenecks showed significant heterozygote excess under the IAM mutation model, and in addition three of those populations showed also heterozygote excess under the TPM model (one tail Wilcoxon tests for heterozygote excess, *P* < 0.05, Table 2). The Grand Isle populations with only marginal signs of a bottleneck (GIP-2011) was one of the populations showing genetic heterogeneity and the presence of a genetic cluster predominantly known from unaffected locations (Fig. 3). This might be a sign of beginning recovery via immigration supported by the fact that this population received 1–1.7% immigrants from CP-2011 and SC-2011, respectively (Table S4).

### Comparison of mating structure between unaffected and oiled populations

Polygamy was the predominant mating strategy based on a comparison of log likelihood values among different possible mating strategies (Table S5 in Supporting Information). Twelve out of the 13 populations were most likely the offspring of polygamy in both sexes. Only one population (SC-2011) had the best support under the monogamy model. The inferred number of mating partners across all 249 inferred parents of both sexes in all 13 populations was on the average 2.06 (*SD* = 1.09). The average number of offspring that each parent contributed in each population was 2.57 (*SD* = 1.56) with a maximum of nine. This number is a minimum estimate since it is based on a limited sample size of offspring. It is meant to be a relative estimate to compare between populations from unaffected and oiled areas, not an absolute measure of fecundity. Sexes could not be distinguished from the inferred parental genotypes and thus male and female specific reproductive contributions are unknown. A considerable proportion of full-sisters (avg. 2.09 ± 2.36%) and half-sisters (16.50 ±5.26%) were frequently caught in the same trap. Detailed results for each population are presented in Table 3.

General linear model analysis showed that condition (unaffected versus oiled populations) significantly influenced the number of parents contributing offspring to the population (*P* = 0.048, *df* = 1, *F* = 5.21), the effective population size (*P* = 0.03, *df* = 1, *F* = 6.75) and the number of offspring per parent (*P* = 0.076, *df* = 1, *F* = 4.02). Condition had also a marginal effect on the number of family clusters (*P* = 0.095, *df* = 1, *F* = 3.48). The factor ‘year’ (2010 vs. 2011 samples) and the combination of ‘year’ * ‘condition’ had no significant influence on any of the dependent variables (all: *P* ≥ 0.15, *df* = 1,9, *F* < 2.52). An additional *t*-test performed for the percentage of fullsib pairs accounting for the unequal variances confirmed the non-significant results from the GLM (*P* = 0.14, *df* = 6.13, *t* = −1.71) as well as the significant results for the number of parents (*P* = 0.028, *df* = 10.99, *t* = 2.54) and effective population size (*P* = 0.03, *df* = 9.99, *t* = 2.55) and the marginal significances for number of offspring per parent (0.053, *df* = 9.84, *t* = −2.20). Number of partners each fly mated with and the percentage of full and halfsibs were not significantly different between unaffected and oiled populations (all: *P* > 0.13, *df* = 1,9, *F* < 2.69). Overall, the effective population size was smaller in oiled populations with fewer parents contributing offspring but with more offspring per mating partner leading to marginally fewer family clusters.

## Discussion

The ecosystems of coastal marshes in general and especially in Louisiana are threatened by multiple human-induced stressors^2^. Oil releases are among the most underreported acute and long-term contamination sources [Gulf Monitoring Consortium (2011), http://skytruth.org/gmc/wp-content/uploads/2012/05/Gulf-Monitoring-Consortium-Report.pdf, date of access: 11/05/2015] and salt marshes are especially vulnerable due to exposure to low tidal wave energy and anoxic conditions allowing oil to persist for years and impact their high bioproductivity^17^,^18^. The aftermath of the greatest accidental marine oil spill in history, the 2010 blowout of the Macondo well in the Gulf of Mexico, provides unprecedented research opportunities for years to come. Tools must be developed not only to assess the impact of this particular oil spill but also to provide screening methods for time and cost-efficient assessments of marsh health after environmental insults to guide remediation efforts.

Surveys of biodegradation by microbes^19^,^20^, plants^21^, and invertebrates^10^ provided valuable insights into immediate effects of oil contamination in the salt marsh after the Deepwater Horizon oil spill. Microbial structure and function shifted to provide efficient hydrocarbon degradation in the marsh^19^. While complete mortality of marsh vegetation was reported in heavily oiled areas, Spartina plants were able to recover from moderate oiling within 7 months^21^. Although the Spartina terrestrial arthropod community and marine invertebrates were initially suppressed by acute oil exposure even in areas where the vegetation seemed intact, the mostly herbivore based feeding guilds had completely recovered a year later^10^. These studies seem to support previous studies showing resiliency of salt marshes towards periodic oil disturbance [e.g.,^18^,^22^,^23^,^24^]. While these observations cause cautious optimism concerning quick marsh remediation, other studies of the Deepwater Horizon oil spill suggest that a deeper look beyond the presence or absence of broad taxonomic groups is necessary to assess sublethal chronic health effects such as genomic, physiological and cardiotoxic damage^6^,^25^, reduced rate of growth^26^, survival and reproduction.

The most vulnerable and, thus, most valuable bioindicators of marsh health are species on the top of the food chain with sediment dwelling developmental stages. We focused our study on the greenhead saltmarsh horse fly, a species usually highly visible and easy to catch, whose development is bound to the sediment of saltmarshes ranging from the Gulf Coast up the Atlantic coast to Nova Scotia^14^. We found not only severe population crashes in oiled areas, but also more subtle effects on population genetic and breeding structure suggesting at least a temporary change in the genetic make-up of oiled populations.

The differences in adult tabanid population estimates between areas unaffected and impacted by oil sampled immediately after the oil spill were dramatic. The apparently immediate decline in adult populations was surprising, since the summer 2010 generation of flies had developed up to 9 months as larvae in the marsh and had emerged prior to the oil spill. Adults of *T. nigrovittatus* do not feed on oiled vegetation and blood meals were available from vertebrates (e.g., birds, cattle and humans). As members of an autogenous species, females do not even need a blood-meal for laying the first clutch of eggs^15^. Therefore, we had initially hypothesized that the adult population would not have been directly exposed to oil in the first year after the oil spill but would reflect the population before the oil spill. This hypothesis was clearly rejected as the adult population crashed in oiled areas immediately after the oil spill.

The most likely reasons for the immediate population crash in oiled areas were the tabanids’ need for fresh water and attraction to oil sheen. Horvath and Zeil^27^ reported that large numbers of different types of insects were found in the hundreds of oil ponds created in the Gulf War in 1991. The authors measured polarization characteristics of crude oil and transparent water surfaces, and showed that the reflected light from oil surfaces was more horizontally polarized, which would create a supernormal stimulus for water-seeking insects. Horvath *et al.*^28^ later showed that crude oil was visually more attractive than water for dragonflies using polaritaxis. Subsequently, Horvath *et al.*^29^ showed that tabanids were attracted by horizontally polarized light. Since adult tabanids in salt marsh environments require fresh water for survival, it is likely that the flies in the oiled areas were attracted to the sheen on the brackish water surface and were trapped due to reduced surface tension of the water. In addition, volatile hydrocarbons might also impact gas exchange, cuticle permeability and membrane structure and function of adults even without direct exposure to oil^30^.

Less surprising than the decline of adult populations in oil impacted areas, but equally dramatic was the difference in larval population estimates for the unaffected vs. oiled marshes. Wilson^31^ showed that approximately one adult tabanid for each tenth of a square meter of larval habitat is produced each generation in flooded hardwood habitats in Louisiana. Based on these data we expected at least 27 larvae to be present in our marsh substrate samples, with the caveat that a direct comparison of the productivity of freshwater and brackish larval habitats might not be entirely accurate. The maximum number of larvae recovered from non-oiled marsh samples (10) fell short of the expected numbers; nevertheless, larval counts in unaffected areas clearly exceeded those in oiled areas, where the majority of sediment samples were entirely devoid of larvae.

Larvae develop as predators and cannibals for 3–9 months in the marsh soil^15^ and are thus indirectly impacted by any decline in the supporting food web. The Deepwater Horizon oil spill caused a drastic reduction in the typically diverse metazoan assemblages that “could eventually translate into long-term effects for higher-level predators and food webs in Gulf ecosystems”^32^. Moreover, sediment-dwelling larvae are directly impacted by toxic soil contamination. For example, Anderson^33^ summarized tabanid population control studies aimed at larval populations that were conducted in the 1950′s and 1960′s using area wide application of chlorinated hydrocarbons. After a single application of insecticide, 100% population suppression ranging for 1–2.5 years was found in several studies, confirming the vulnerability of tabanids larvae to soil contamination and the severe and lasting impact on tabanid populations.

Subsurface water in Louisiana marshes was shown to have significant levels of polycyclic aromatic hydrocarbons (PAH) and acute toxicity to killifish immediately after the oil spill^6^. Chronic genomic and physiological changes in fish were also observed^6^,^7^. Whitehead *et al.*^6^ reported that the PAH levels in subsurface water in previously oiled Louisiana marshes remained high enough to have biological effects on killifish for up to two months, but they also provided data showing that high levels of oil were retained in the sediment of oiled marsh at their last sample taken at five months post intrusion.

We suggest that the initial crashes in adult tabanid populations were associated with acute adult mortality, but that the sustained tabanid population suppression was due to toxic effects directly to larval tabanids and/or important elements of their food web. Therefore, this top predator species with sediment-dwelling larvae did not show the quick recovery seen from other insects, such as the community that is built around Spartina herbivores^10^.

The apparently severe population crash and reduced effective population size of tabanid adults and larvae in oiled areas led to genetic bottlenecks in all but one of the oiled populations. The bottleneck test based on faster reduction of allele numbers relative to reduction in heterozygosity is actually considered having little power and requires that extreme bottlenecks have occurred in recent times to show significance, especially in the first generations experiencing the bottleneck^34^. This result, together with the crash in population numbers underscores the severity of the recent impact on oiled populations.

Whether these findings translate into reduced evolutionary potential of populations depends on remediation success of oiled marsh sites and recovery of a sustaining food web. Theory^35^ and some empirical studies^36^ have suggested that the consequences of severe bottlenecks may be mitigated if subsequent recovery is quick, while populations that recover more slowly [e.g.,^37^,^38^] suffer loss of fitness.

While substantial loss of genetic diversity, increased linkage disequilibrium, reduced heterozygosity and correlative effects on fitness via inbreeding depression are frequently discussed as observed or inferred consequences of genetic bottlenecks^39^, few studies exist exploring the relationship between genetic architecture of a population experiencing bottlenecks and breeding structure. Our study showed decreased effective population size, number of breeders, and family clusters in oiled populations that experienced genetic bottlenecks compared to populations in unaffected areas (Table 4). The severe population crash caused fewer parents to be available in oiled populations to contribute offspring. It is also likely that reduced food supply in form of soil metafauna for the predatory larvae and blood meals for adults in oiled areas negatively affects larval survival and adult fecundity^15^. Thus, the effective population size will probably continue to be decreased for several generations until immigration replenishes the gene pool.

Gene flow and migration rates among unaffected populations were slightly higher than among oiled populations. This was not due to geographical distance since the distance between oiled populations was actually smaller and no isolation by distance effects were detected within 150 km, i.e., within the range of distance among unaffected (max. 144 km) and among oiled populations (max. 45 km). Interestingly, there was a directional bias in migrations rates with emigration rates being marginally lower from oiled populations, but immigration into oiled populations were equal to those into unaffected populations. This suggests at least a temporary negative effect of oil contamination on dispersal. Other studies showed that sublethal exposure to crude oil causes changes in heart function^25^ and shape, leading to a significant reduction in swimming performance in fish which likely would result in decreased dispersal ability^7^, but no prior studies have been conducted concerning insect dispersal abilities.

The Deepwater Horizon spill affected over 700 km of marsh shoreline in Louisiana alone with the peak amount of oiling in July 2010; but almost 200 km still displayed some degree of oiling after two years and more^40^. However, oil contamination was patchy across locations and time leaving healthy areas as likely sources for repopulation. The absence of isolation by distance in adult dispersal, the comparatively unrepressed immigration rates into oiled populations found in this study, and the high reproductive capacity of single females even without blood meals^15^ should allow for a recovery in census numbers. Indeed, there were site specific results that indicated potential recovery of tabanid populations at two sites at the Grand Bayou location where 21–24 flies per hour were captured in August 2011, which is an approximately five fold increase compared to the previous catches (Table S1). One day surveys at previously sampled trap sites in June 2012 and 2014, including Ship Channel and Rockefeller Wildlife Refuge (unaffected by oil) and Elmer’s Isle, Grand Isle and Grand Bayou (oiled) also show signs of recovery in census numbers of the previously oiled populations. However, horse fly numbers in oiled populations were still lower than in unaffected populations. Thus, the top predator levels recover much slower than the herbivorous insect community^10^.

Continued observation of the fate of the tabanids populations in the oiled areas will shed light on population viability. Genetic studies and larval surveys need to continue to discern whether census increase is based mostly on immigrants or if the marsh sediment has detoxified sufficiently to allow local development of larvae. The latter would be a valuable indicator sign of ecosystem recovery.

## Material and Methods

### Collections

Collection permits for horse fly larvae and adults were obtained from the Louisiana Department of Wildlife and Fisheries (permit numbers LNHP-10-074 and LNHP-11-092). Adult female flies were collected every other week from June through October in 2010 and 2011, at four locations. Each location had 4–5 trap sites separated by >1km (Fig. 1). Since we had no accurate prediction where the oil would make landfall, the locations were selected based upon the investigator’s (LF) knowledge of prior large populations of greenhead horse flies. The sites were selected relative to available access, absence of confined livestock, and all within view of Spartina marshes. Cameron Parish and Cypremort Point in St Mary’s Parish in West Louisiana were not impacted by the oil spill (unaffected controls). However, oiling was reported at Elmer’s and Grand Isle on May 24, 2010 and at Grand Bayou on June 18, 2010 [The New York Times (2005), http://www.nytimes.com/interactive/2010/05/01/us/20100501-oil-spill-tracker.html, date of access: 11/05/2015].

Tabanids were captured with canopy traps baited with dry ice^41^. Only females are attracted to CO_2_ and thus the samples contained exclusively females. The exact daily trapping period (ranging from 2–8 hours) was recorded for each site at each location, and the highest three trap counts were used for analysis. The captured flies were transferred on dry ice and stored at −80 °C. Specimens were identified as *T. nigrovittatus* using the methods of Sofield *et al.*^42^, counted using a dissecting microscope and cold plate, and returned to −80 °C for storage. Voucher specimens were deposited in the Louisiana State Arthropod Museum at the Department of Entomology of the Louisiana State University Agricultural Center.

A subset of 13 samples of all that were collected for population abundance analyses were used for population genetic studies. For microsatellite genotyping, we used horse flies that were collected in June 2010 and again in June 2011 from single traps representing three different trap sites in the unaffected marshland along the coast of Western Louisiana (Ship Channel, Rockefeller Wildlife Refuge, and Cypremort Point, Fig. 1). Due to low trap catches in oiled locations in Eastern Louisiana (Grand and Elmer’s Isle and Grand Bayou) in both years, we genotyped flies from the traps with the highest fly catch.

We also evaluated the presence or absence of tabanid larvae from 5–7 spartina marsh substrate samples collected between 8/25/2011 and 9/28/2011 at Rockefeller Wildlife Refuge near adult collection sites (ACS), Cypremort Point at sites near ACS 1 and 2, Grand Isle near all ACS, and Grand Bayou near all ACS (Fig. 1) using the techniques of Dukes *et al.*^43^. Marsh samples were collected near adult collection sites by excavating 5 m long 0.5 m wide 10 cm deep transects. Tabanid larvae were removed from the sediment samples using a brine-flotation system, and the collected larvae were stored in 95% ethanol.

### Microsatellite genotyping

Total DNA was extracted from thoraces of horse flies using the DNeasy Tissue Kit (Qiagen Inc., Valencia, CA). Up to 30 horse flies per sample were genotyped at ten polymorphic loci. A detailed description of the loci selection process during which unreliable loci (e.g., with high null allele frequencies) were identified and omitted from population genetic analyses, polymerase chain reaction and genotype scoring procedures can be found in Husseneder *et al.*^16^. Summary statistics, i.e., locus characteristics, GenBank accession numbers, allele numbers, inbreeding coefficients and observed versus expected heterozygosity (gene diversity) can be found in Table S6 in Supporting Information.

### Statistical analysis of adult horse fly abundance

Exploratory analyses indicated that data were not normally distributed. The average numbers in each trap collection were transformed to y = log(x+1) to normalize prior to statistical analysis. First, a two way ANOVA (PROC MIXED, SAS Institute 2010) was used to compare log transformed tabanid catch data across years and locations. Model main effects were Year (2010, 2011) and Region (Plaquemines, Jefferson, St. Mary, and Cameron). Second, a three way ANOVA was performed to add Month (June, July, August, September, October) to the previous main model effects. All possible interactions among the main effects in the model, and a Tukey Kramer adjustment (α = 0.05) were used to separate the means.

### General population genetic statistics

Samples from different traps were tested for significant genotypic differentiation using pairwise log-likelihood *G*-Statistics with standard Bonferroni corrections at a 0.001 nominal level for multiple comparisons (FSTAT^44^). Genetically separated samples (populations) were then used as population genetic units of analysis. Allelic richness was calculated using the sample-size independent rarefaction analysis as implemented in FSTAT^44^,^45^. Observed heterozygosity was calculated for each locus in each population using GDA^46^ and compared to gene diversity^47^ as a measure of expected heterozygosity. *F*-statistics were calculated using the methods of Weir and Cockerham^48^ in FSTAT with *F*_IT_ representing the standard coefficient of individuals relative to the total population, *F*_IS_ representing inbreeding in individuals relative to the population and *F*_ST_ representing the genetic differentiation among populations. Standard errors (*SE*) were obtained by jackknifing over loci. Tests for deviation from Hardy-Weinberg-Equilibrium were performed with FSTAT for each locus and population based on randomizing alleles within populations and calculating *F*_IS_. Statistics such as Kolmogorov-Smirnov tests for normality, parametric (*t*-tests) and nonparametric tests (Kruskal-Wallis, Mann-Whitney-U) and well as General Linear Models were performed with SPSS (IBM Corp. Released 2012. IBM SPSS Statistics for Windows, Version 21.0. Armonk, NY: IBM Corp.). *P*-values ≤ 0.05 were considered significant and *P*-values up to 0.10 were considered marginal.

### Population genetic structure and isolation by distance

To obtain ad hoc approximations of the number of genetic clusters in the total population and the genetic relationships among populations, individual flies were probabilistically assigned to genetic clusters based on their multilocus genotypes using TESS 3.1^49^. The simulations were run 10 times without admixture for each Kmax ranging from 2–15 for 5,000 sweeps with 500 sweeps burn-in. The maximum number of genetic clusters (Kmax) was determined from changes in the deviance information criterion (DIC^50^, Figure S1). Results from replicates were averaged using CLUMPP 1.1.2^51^. Finally, the estimated membership coefficients of each individual multilocus genotype to each genetic cluster were plotted with STRUCTURE PLOT^52^.

Isolation by distance among populations was determined by transforming genetic distance *F*_ST_ into *F*_ST_/(1 − *F*_ST_) and performing a regression against the natural logarithm of geographical distance. Matrix correlations were tested for significance using Mantel tests (one sided *P*-values from 1,000 randomizations, IBD v. 1.52^53^).

In addition, we employed Bayesian statistics using Markov Chain Monte Carlo methods (BayesASS v. 1.3) to estimate recent gene flow (*m* = proportion of migrants), because these techniques allow for deviation from migration-drift balance and Hardy-Weinberg equilibrium^54^. Preliminary runs were used to assure that delta values for allele frequency, migration rate and inbreeding stayed between 20–60% of the total chain length^54^. The final settings were 3 million iterations, a sample frequency of 2000, a burn-in period of 999999 and delta values of 0.25.

### Detection of genetic bottlenecks

Bottlenecks can be detected based on the fact that a bottleneck reduces allele numbers faster than heterozygosity. Genotypes of individuals in each population were tested for heterozygote excess across loci using a Wilcoxon sign-rank test under different mutation models (IAM, i.e., infinite allele model, TPM, i.e., two-phased model of mutation, SMM, i.e., stepwise mutation model), as implemented in BOTTLENECK v. 1.2.02^55^.

### Mating structure, sibship and effective population sizes

Pedigree structure in each population was inferred using the full pedigree likelihood method implemented in COLONY v.2.0.3.1^56^ based on multilocus genotypes. COLONY was chosen for sibship evaluation because previous analyses revealed considerable inbreeding and relatedness possibly due to incest in tabanids^16^ and the full-pedigree likelihood method in COLONY is only slightly affected by relatedness and the mating system in the population^57^.

To infer the best configuration of relationship structure, populations of offspring (trap samples) were subjected to full maximum likelihood calculations^56^,^58^. Since larvae develop 3–9 months the females caught in traps were most likely offspring without mothers present in the dataset and thus high relatedness among individuals reflects sibship (sisters) and not mother-daughter relationships. Length of run was set as medium and number of runs was 1, which typically suffices for most datasets^56^. The program option to update allele frequencies during the process of searching for the maximum likelihood configuration was not chosen since family sizes were unknown, possibly large (compared to sample size) and variable^56^. Allelic dropout rate was set to zero as previously confirmed by MICRO-CHECKER analyses^16^ and error rates were set to 0.005 for the computations.

To identify the predominant mating strategy in each population independent runs were performed specifying the three possible mating strategies (both sexes monogam or polygam, one sex monogam and the other polygam) allowing the occurrence of inbreeding. Log likelihood values for the best pedigree configuration based on the observed genotypes were compared among the hypothesized mating strategies. Only results for the mating strategy model with the best fit (polygamy) were presented in the study.

Based on the Best Maximum Likelihood Configuration output of the program, we determined the number of inferred parents contributing offspring (i.e., a measure of the breeding population size) and the number of family clusters in each population. Obtained values were corrected for uneven sample sizes by calculating each for a sample size of 30 individuals genotyped per population. We also estimated the number of partners that individuals of both sex mated with and the number of offspring produced per parent. The percentages of fullsib and halfsib pairs in each population were inferred from sibship assignment plots in COLONY.

Effective population size (*N*_e_) was inferred with the sibship assignment method (i.e. via frequencies of a pair of randomly sampled offspring sharing one or both parents) implemented in COLONY^56^ rather than the heterozygosity based methods^59^, because inbreeding in the tabanid life cycle^16^ and the potential bottlenecks suffered by oiled populations would likely bias heterozygosity based algorithms. The sibship assignment method proved to be more accurate than other methods for “real-world” datasets since it does not assume isolated populations or random mating and performs comparatively well even with small sample sizes and numbers of loci^60^. Note that the actual number of breeders is not necessarily equal to *N*_e_ since it is dependent on the variance in family size^60^.

### Analysis of variance of population genetic parameters

Two-Factor Analysis of Variance was performed using General Linear Model Multivariate tests (SPSS) to investigate whether the factors “year” (2010 versus 2011) and/or “condition” (unaffected versus oiled populations) influenced the dependent variables measured to determine population and mating structure. Significant deviations from equal error variances were detected in the number of offpring per parent for the factor ‘condition’ (Levene’s *P* = 0.03, *df* = 1,11, *F* = 6.18) and with marginal significance when all factors and combinations were considered (*P* = 0.10, *df* = 3,9, *F* = 2.79). Log transformation removed the issue (*P* > 0.20). Unequal variances were also detected in the percentage of fullsib pairs (Levene’s test: *P* = 0.001, *df* = 3,9, *F* = 14.85) when all factors and combinations were considered, but not for ‘condition’ (*P* = 0.15, *df* = 1,11, *F* = 2.48). Transformations failed to remove unequal error variance in this dependent variable. Thus, an additional independent samples *t*-test not assuming equal variances was performed.

## Additional Information

**Data availability**: Data are publicly available through the Gulf of Mexico Research Initiative Information & Data Cooperative (GRIIDC). Detailed sample information including GPS location, dates, trap times and horse fly counts and can be found at https://data.gulfresearchinitiative.org/data/R3.x169.000:0001/ in the dataset by LF: Adult tabanid population data and available voucher specimens (doi:10.7266/N75718ZM). Raw data of microsatellite genotypes are available at https://data.gulfresearchinitiative.org/data/R3.x169.000:0002/ in the dataset by CH: *Tabanus nigrovittatus* microsatellite data for the assessment of population genetics of 13 populations sampled from coastal Louisiana, 2010-2011 (doi:10.7266/N71J97N2).

**How to cite this article**: Husseneder, C. *et al.* Impact of the 2010 Deepwater Horizon oil spill on population size and genetic structure of horse flies in Louisiana marshes. *Sci. Rep.* **6**, 18968; doi: 10.1038/srep18968 (2016).

## Supplementary Material

Supplementary Information

## Figures and Tables

**Table 1:** Mean number of *Tabanus nigrovittatus* trapped (flies/hour) by region in 2010 and 2011.

Region	2010		2011	
	Mean ± SE	Mean (log x+1) ± SE	Mean ± SE	Mean (log x+1) ± SE
Cameron Parish	82.24 ± 6.62	3.88 ± 0.17^a^	53.25± 6.92	3.35 ± 0.18^ab^
St. Mary Parish	37.97 ± 6.64	2.62 ± 0.17^b^	38.01± 6.49	3.32 ± 0.17^ab^
Jefferson Parish	0.85 ± 6.06	0.42 ± 0.16^d^	1.34 ± 6.46	0.62 ± 0.17^cd^
Plaquemines Parish	3.94± 5.82	1.17 ± 0.15^c^	4.51± 5.43	1.13± 0.14^c^

Letters ^a, b, c, d^ indicate statistical differences: counts with different letters are statistically different (P < 0.05; Tukey-Kramer).

**Table 2:** Probabilities to reject mutation-drift equilibrium due to heterozygote deficiency or heterozygote excess (genetic bottleneck) for three different mutation models (IAM = infinite allele model, TPM = two-phase mutation model, SMM = stepwise mutation model) in unaffected and oiled tabanids populations.

	Pristine						Oiled						
	SC-2010	CP-2010	RWR-2010	SC-2011	CP-2011	RWR-2011	GI-2010	EI-2010	GB2-2010	GIW-2011	GIP-2011	GB2-2011	GB3-2011
One tailed Wilcoxon test for heterozygote deficiency													
IAM	0.05	>0.20	>0.20	0.14	>0.20	0.007	>0.20	>0.20	>0.20	>0.20	>0.20	>0.20	>0.20
TPM	0.007	>0.20	>0.20	0.012	>0.20	0.002	>0.20	>0.20	>0.20	>0.20	>0.20	>0.20	>0.20
SSM	0.001	0.039	>0.20	0.004	0.027	0.001	>0.20	>0.20	>0.20	>0.20	>0.20	>0.20	>0.20
for heterozygote excess													
IAM	>0.20	>0.20	>0.20	>0.20	>0.20	>0.20	0.012	0.037	0.008	>0.20	0.082	0.002	0.019
TPM	>0.20	>0.20	>0.20	>0.20	>0.20	>0.20	0.08	>0.20	0.045	>0.20	>0.20	0.009	0.15
SSM	>0.20	>0.20	>0.20	>0.20	>0.20	>0.20	>0.20	>0.20	0.15	>0.20	>0.20	>0.20	>0.20

**Table 3:** Mating structure of unaffected and oiled populations of *T. nigrovittatus.*

	Pristine						Oiled							Average across Populations			
	SC-2010	CP-2010	RWR-2010	SC-2011	CP-2011	RWR-2011	GI-2010	EI-2010	GB2-2010	GIW-2011	GIP-2011	GB2-2011	GB3-2011	Pristine 2010	Pristine 2011	Oiled 2010	Oiled 2011
No. parents	26	24	31	23	32	24	16	18	27	23	26	18	17	27.00	26.33	20.33	21.02
SD														3.6	4.93	5.85	4.27
N_**e**_	26	26	38	15	27	24	9	19	24	13	24	20	16	30	23	17.33	18.25
Range/SD	(15–50)	(15–50)	(23–68)	(9–33)	(14–57)	(14–46)	(4–24)	(11–38)	(13–50)	(6–31)	(13–47)	(11–38)	(8–34)	6.93	4.58	7.64	4.79
No. partners	2.08	2.08	1.74	2.5	1.71	2.17	1.75	1.75	1.89	2.21	1.6	2.67	2.53	1.97	2.13	1.79	2.25
SD	1.20	1.14	0.86	1.40	0.78	1.13	0.71	0.71	0.83	1.12	0.88	1.24	1.23	0.19	0.39	0.08	0.47
No. offspring/partners	2.31	2.5	1.94	3	1.9	2.5	3.75	3.75	2.11	2.57	2.3	3.33	3.53	2.25	2.47	3.2	2.93
SD	1.49	1.38	1.09	2.38	1	1.56	1.28	1.28	0.9	1.65	1.17	1.46	2.03	0.28	0.55	0.95	0.59
No. family clusters	5.00	5.00	6.00	3.00	4.50	2.00	4.00	2.00	3.16	3.33	5.22	3.00	2.00	5.33	3.33	3.00	3.25
														0.58	1.52	1.00	1.25
% fullsibs	0.69	1.15	0.69	1.27	1.05	1.38	9.52	1.15	1.17	1.31	3.16	1.61	2.99	0.84	1.23	3.95	2.27
SD														0.27	0.17	1.00	1.25
% halfsibs	14.02	13.1	9.2	20.92	12.63	14.02	25.71	18.39	14.04	25.71	10.67	17.01	19.08	12.1	15.86	19.38	18.55
SD														2.56	4.44	5.89	6.92

Variables for all populations were inferred via full likelihood pedigree analyses as implemented in COLONY. Averages for unaffected and oiled populations collected in 2010 and 2011 were derived from descriptive statistics in SPSS.

No. parents = number of parents producing offspring inferred from offspring genotypes (effective number of breeders). *N*_e_ = Effective population size and its range as inferred from the sibship assignment method in COLONY. No. partners = average number of mates per individual. No. offpring/parent = average number of offspring a parent contributes to the trap sample. No. family clusters = number of groups with individuals linked by pedigree. % fullsibs and % halfsibs = percentage of total number of pairs being full sisters or half sisters. *SD = *standard deviation. For comparison among populations No. parents and No. family clusters were sample size corrected by extrapolating for the maximum sample size (*n* = 30).

**Table 4:** Summary of the differences between unaffected and oiled populations of tabanids.

	Not oiled	Oiled
Adult fly counts	High	Low
Larvae recovered from marsh soil	High	Low
Effective population size	High	Low
Number of breeders	High	Low
Number of families	High	Low
Number of migrants, gene flow	High	Low
Genetic bottlenecks	No	Yes
