# Supplementary material for: Impact of the 2010 Deepwater Horizon oil spill on population size and genetic structure of horse flies in Louisiana marshes
Source: Sci Rep. 2016 Jan 12;6:18968. doi: 10.1038/srep18968 (PMC4709594; doi:10.1038/srep18968)

Supplementary Information

Impact of the 2010 Deepwater Horizon oil spill on population size and genetic structure of horse flies in Louisiana marshes

CLAUDIA HUSSENEDER, JENNIFER R. DONALDSON, AND LANE D. FOIL

Supplementary Tables

Table S1: Mean number of *T. nigrovittatus* trapped (flies/hour) by month and region.

Since year had no effect on fly counts (Table 1), numbers were averaged across 2010 and 2011.
Letters a, b, c indicate statistical differences: counts with different letters are statistically different ($P < 0.05$; Tukey-Kramer).

Region	June		July		August		September		October	
	Mean ± SE	Mean (log x+1) ± SE*	Mean ± SE	Mean (log x+1) ± SE*	Mean ± SE	Mean (log x+1) ± SE*	Mean ± SE	Mean (log x+1) ± SE*	Mean ± SE	Mean (log x+1) ± SE
Cameron	120.55 ± 10.26	4.07 ± 00.26 ^{a}	57.44 ± 08.23	3.48 ± 00.21 ^{a}	89.70 ± 11.98	4.34 ± 00.31 ^{a}	23.31 ± 10.62	2.89 ± 00.27 ^{a}	47.72 ± 11.98	3.30 ± 00.31 ^{a}
St. Mary	89.53 ± 10.94	4.14 ± 00.28 ^{a}	41.04 ± 07.73	3.12 ± 00.20 ^{a}	24.80 ± 10.38	2.95 ± 00.27 ^{a}	22.16 ± 10.38	2.82 ± 00.27 ^{a}	12.44 ± 11.98	1.84 ± 00.31 ^{ab}
Jefferson	2.27 ± 10.26	0.84 ± 00.26 ^{b}	0.40 ± 10.62	0.25 ± 00.27 ^{b}	0.62 ± 09.28	0.44 ± 00.24 ^{b}	1.44 ± 09.02	0.61 ± 00.23 ^{b}	0.72 ± 10.26	0.46 ± 00.26 ^{bc}
Plaquemines	6.57 ± 07.26	1.85 ± 00.19 ^{b}	4.66 ± 07.87	1.26 ± 00.20 ^{b}	4.27 ± 09.78	1.24 ± 00.25 ^{b}	5.34 ± 06.47	1.22 ± 00.17 ^{b}	0.29 ± 11.98	0.18 ± .31 ^{c}

Table S2: Population genetic statistics for 13 pristine and oiled tabanids populations. A (r) = Allelic richness based on rarefaction [45]; *SE* = standard error; H_o = observed heterozygosity, H_S = gene diversity [47] as a measure of expected heterozygosity, F_{IS} = coefficient of inbreeding, *t*-test ≠ 0 = two-tailed one-sample *t*-test for F_{IS} values different from zero across 10 loci.

**Area**	**Population**<br>**Sample size**	**A (r)**<br>***SE***	H_O<br>H_S	F_{IS}<br>***SE***	***t*-test ≠ 0**<br>***P***
**Unaffected by the oil spill**	**SC-2010**<br>30	3.19<br>0.23	0.25<br>0.33	0.22<br>0.05	0.09
	**CP-2010**<br>30	2.68<br>0.30	0.23<br>0.31	0.23<br>0.06	0.11
	**RWR-2010**<br>30	2.98<br>0.25	0.20<br>0.37	0.38<br>0.07	0.02
	**SC-2011**<br>30	2.74<br>0.27	0.21<br>0.27	0.17<br>0.04	0.11
	**CP-2011**<br>20	2.41<br>0.32	0.17<br>0.27	0.38<br>0.06	0.01
	**RWR-2011**<br>29	2.77<br>0.27	0.13<br>0.24	0.32<br>0.07	0.04
**Oiled**	**GI-2010**<br>15	2.80<br>0.21	0.37<br>0.49	0.33<br>0.16	0.12
	**EI-2010**<br>30	2.55<br>0.24	0.30<br>0.35	0.14<br>0.07	0.31
	**GB-2010**<br>19	2.19<br>0.23	0.20<br>0.35	0.44<br>0.10	0.03
	**GIW-2011**<br>18	2.37<br>0.22	0.33<br>0.33	0.04<br>0.09	0.79
	**GIP-2011**<br>23	3.90<br>0.46	0.26<br>0.50	0.43<br>0.08	0.01
	**GB2-2011**<br>26	3.25<br>0.40	0.33<br>0.48	0.27<br>0.07	0.08
	**GB3-2011**<br>30	2.52<br>0.22	0.33<br>0.37	0.11<br>0.06	0.40

Table S3: Pairwise genetic distance (F_{ST}) between 13 tabanid populations collected from oiled and unaffected areas.

	Locations Unaffected By Oil						Oiled Locations						
F_{ST}	SC-2010	SC-2011	CP-2010	CP-2011	RWR-2010	RWR-2011	GI-2010	GIW-2011	GIP-2011	EI-2010	GB2-2011	GB3-2011	GB-2010
**SC-2010**	-	0.235	0.074	0.122	0.048	0.338	0.370	0.534	0.308	0.413	0.254	0.421	0.399
**SC-2011**		-	0.181	0.083	0.108	0.327	0.440	0.588	0.251	0.489	0.239	0.503	0.455
**CP-2010**			-	0.113	0.062	0.389	0.383	0.551	0.301	0.431	0.239	0.423	0.384
**CP-2011**				-	0.042	0.318	0.409	0.563	0.239	0.452	0.231	0.466	0.419
**RWR-2010**					-	0.268	0.353	0.488	0.212	0.392	0.200	0.407	0.358
**RWR-2011**						-	0.472	0.542	0.256	0.460	0.311	0.481	0.405
**GI-2010**							-	0.224	0.199	0.279	0.143	0.129	0.222
**GIW-2011**								-	0.186	0.332	0.281	0.164	0.336
**GIP-2011**									-	0.294	0.128	0.238	0.237
**EI-2010**										-	0.185	0.277	0.275
**GB2-2011**											-	0.201	0.173
**GB3-2011**												-	0.210
**GB-2010**													-

Table S4: Proportions of migrants from and into each population

Values in bold represent the proportions of individuals derived from the source populations. Migration rates > 5% are italicized. Standard deviations for all distributions were <0.03.

From	SC-2010	SC-2011	CP-2010	CP-2011	RWR-2010	RWR-2011	GI-2010	GIW-2011	GIP-2011	EI-2010	GB2-2011	GB3-2011	GB2-2010
Into													
SC-2010	***0.680***	0.005	*0.280*	0.005	0.003	0.003	0.003	0.003	0.004	0.004	0.003	0.003	0.003
SC-2011	0.001	***0.987***	0.001	0.001	0.001	0.001	0.001	0.001	0.001	0.001	0.001	0.001	0.001
CP-2010	0.001	0.001	***0.988***	0.001	0.001	0.001	0.001	0.001	0.001	0.001	0.001	0.001	0.001
CP2011	0.001	0.002	0.002	***0.982***	0.002	0.002	0.001	0.002	0.002	0.001	0.001	0.002	0.002
RWR-2010	0.004	0.007	*0.109*	*0.173*	***0.677***	0.004	0.004	0.004	0.005	0.004	0.003	0.004	0.004
RWR-2011	0.003	0.003	0.003	0.004	0.003	***0.677***	0.003	0.006	0.028	0.007	0.005	0.003	0.005
GI-2010	0.004	0.022	0.006	0.008	0.004	0.004	***0.923***	0.005	0.004	0.004	0.008	0.004	0.005
GIW-2011	0.002	0.003	0.003	0.003	0.002	0.002	0.002	***0.968***	0.005	0.003	0.002	0.002	0.002
GIP-2011	0.004	0.017	0.005	0.010	0.003	0.003	0.004	*0.108*	***0.828***	0.004	0.005	0.004	0.005
EI-2010	0.001	0.001	0.001	0.001	0.001	0.001	0.001	0.001	0.001	***0.989***	0.001	0.001	0.001
GB2-2011	0.003	*0.073*	0.003	0.004	0.003	0.003	0.003	0.003	0.004	0.003	***0.894***	0.003	0.004
GB3-2011	0.003	0.005	0.005	0.006	0.003	0.002	0.003	*0.261*	0.005	0.003	0.026	***0.677***	0.004
GB2-2010	0.001	0.001	0.001	0.001	0.001	0.001	0.001	0.001	0.001	0.001	0.001	0.001	***0.985***

Table S5: Log likelihood values calculated for possible mating strategies in tabanid populations.
Bold values indicate the most likely mating strategy (i.e., polygamy in 12 of the 13 populations)

Mating Strategy	Pristine Populations						Oiled Populations						
	SC-2010	CP-2010	RWR-2010	SC-2011	CP-2011	RWR-2011	GI-2010	EI-2010	GB2-2010	GIW-2011	GIP-2011	GB2-2011	GB3-2011
Both sexes monogamy	-296	-251	-331	**-195**	-159	-222	-123	-235	-163	-137	-315	-325	-261
One sex monogamy, other polygamy	-285	-244	-321	-217	-152	-215	-120	-227	-162	-133	-306	-318	-256
Both sexes polygamy	**-268**	**-234**	**-306**	-225	**-146**	**-199**	**-113**	**-209**	**-157**	**-126**	**-292**	**-301**	**-238**

Table S6: Summary statistics for the 10 polymorphic loci used for microsatellite genotyping of *T. nigrovittatus*.

Details concerning primer sequences, loci selection and PCR protocols can be found in Husseneder et al. (2014).

N_A per pop: allele count per locus and population; N_A total: allele count across all 13 populations in the dataset; F_{IS}: coefficient of inbreeding within populations; *SE*: standard error derived from jackknifing over populations (FSTAT); H_O: observed heterozygosity and H_S: gene diversity [47] as a measure of expected heterozygosity.

Locus	Repeat	GenBank #	N_A per pop<br>N_A total	F_{IS}<br>*SE*	H_O<br>H_S
X18	(TG)^3(AGAT)^6	JN547239	2-9<br>14	0.47<br>0.10	0.30<br>0.60
BA9	(AGAT)^4	JN547241	2-7<br>11	0.65<br>0.05	0.12<br>0.38
2Y8	(ACAT)^6	JN547240	2-6<br>7	0.23<br>0.04	0.35<br>0.49
VDT	(GTT)^5	JN547244	1-5<br>6	0.37<br>0.12	0.27<br>0.42
7JU	(AAC)^4	JN547251	1-2<br>2	-0.17<br>0.08	0.29<br>0.20
A7Y	(AAC)^5	JN547245	1-4<br>4	0.38<br>0.14	0.18<br>0.29
42Z	(ACT)^4	JN547246	1-3<br>3	0.30<br>0.07	0.17<br>0.28
CFO	(GT)^6	JN547256	1-2<br>2	0.17<br>0.21	0.03<br>0.05
H82	(GT)^7	JN547253	2-7<br>10	0.32<br>0.05	0.32<br>0.49
YN9	(AT)^9	JN547255	1-5<br>7	-0.11<br>0.07	0.48<br>0.43

Supplementary Figures

Figure S1: Plot of deviance information criterion (DIC)

The DIC curve decreases sharply up to Kmax = 6 and then levels off. The dataset is best described assuming the maximum number of clusters being six.

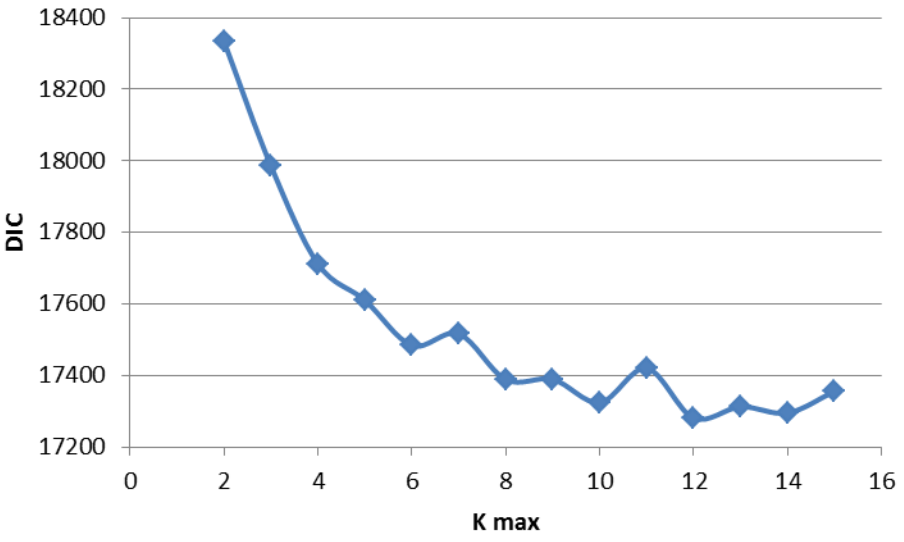

Figure S2. Correlation of genetic distance and geographic distance. Weak, but significant isolation by distance over a large scale, but no isolation by distance among populations within 150 km.

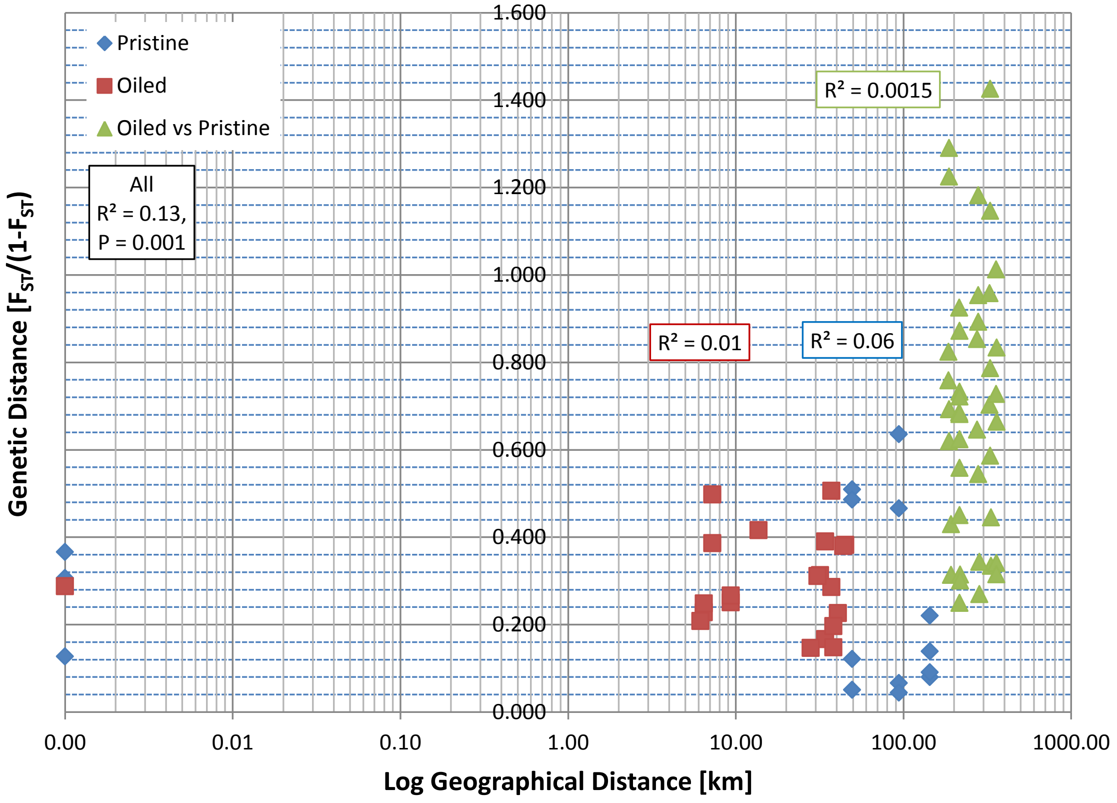

Supplement: Supplementary Information [file srep18968-s1.pdf]
